# Cancer, Mortality, and Acute Kidney Injury among Hospitalized Patients with SARS-CoV-2 Infection

**DOI:** 10.31557/APJCP.2021.22.2.517

**Published:** 2021-02

**Authors:** Johnathan A Khusid, Adan Z Becerra, Blair Gallante, Areeba S Sadiq, William M Atallah, Ketan K Badani, Mantu Gupta

**Affiliations:** 1 *Department of Urology, Ichan School of Medicine at Mount Sinai, New York, NY, USA. *; 2 *Department of Surgery, Rush University Medical Center, Chicago, IL, USA. *

**Keywords:** COVID-19, bladder cancer, kidney cancer, prostate cancer, acute kidney injury

## Abstract

**Background:**

To evaluate Coronavirus Disease 2019-(COVID19) patients treated within our academic medical system to determine if history of malignancy, both in general and specifically in genitourinary oncology patients, is associated with adverse clinical outcomes, including acute kidney injury (AKI) and mortality.

**Methods:**

We conducted a retrospective cohort study among patients with confirmed severe acute respiratory syndrome coronavirus 2 (SARS-CoV-2) infection in a multi-hospital, academic medical institution in New York City. Outcomes included mortality, intensive care unit (ICU) admission and AKI among hospitalized patients. We also evaluated risk of hospitalization among all patients with SARS-CoV-2 infection. Multilevel logistic regression models were used for analysis.

**Results:**

We identified 6,893 patients who met inclusion criteria, of which 4,018 were hospitalized. Among hospitalized patients 374 (9%) had a history of cancer, 281 (7%) experienced AKI, and 1,045 (26%) died. In adjusted analyses, patients with a history of cancer had 1.33 (95% CI = 1.05, 1.69) times the odds of death compared to those without cancer and this appeared to be driven by lung cancer (odds ratio (OR) = 2.44, 95% CI= 1.05, 4.39). Patients with a history of genitourinary cancer were not at higher risk of mortality compared to those without cancer (OR=0.99, 95% CI= 0.61, 1.62). History of cancer was not associated with ICU admission or AKI in overall and subgroup analyses.

**Conclusions:**

Patients with a history of cancer who are hospitalized with SARS-CoV-2 infection are not at greater risk for AKI, though they are at higher risk for mortality as compared to patients without a history of cancer. The increased risk in mortality appears driven by patients with pulmonary neoplasms. Patients with a history of genitourinary malignancies do not appear to be at higher risk for AKI or for mortality compared to the general population.

## Introduction

The Coronavirus Disease-2019 (COVID19) pandemic has led to radical shifts in the delivery of healthcare. To minimize the risk of person-to-person transmission, particularly in the nosocomial setting, attempts have been made to increase utilization of telehealth(Wosik et al., 2020). Yet, many aspects of healthcare require in-person interactions. This is particularly true for urologic oncology patients as chemotherapy infusions, radiation therapy, surgical expatriation, and radiologic surveillance are components of routine care. Furthermore, the time-sensitive nature of malignancy makes delays in care problematic for many patients. 

To this end, several collaborative reviews have been published on strategies for the appropriate triage of patients with urologic malignancy during the COVID19 pandemic(Goldman and Haber, 2020; Stensland et al., 2020; Wallis et al., 2020). These recommendations are largely based on the existing literature regarding the natural history of individual urologic malignancies. For highly aggressive malignancies such as muscle invasive bladder cancer, significant delays in care are clearly unacceptable(Russell et al., 2020). Similarly, for those malignancies with a more indolent behavior such as low-risk prostate cancer, delays and modifications to routine care are unlikely to produce significant adverse outcomes(van den Bergh et al., 2013). However, for moderately aggressive malignancies such as intermediate-risk and high-risk prostate cancer, non-muscle invasive bladder cancer, and most kidney cancers, the risks and benefits of delaying and modifying care should be based both on the natural history of the cancer, and the natural history of COVID19 in these patients and cancer patients in general. 

Indeed, several large population-based studies from various countries have suggested malignancy is associated with adverse outcomes in COVID19 patients(Berenguer et al., 2020; Liang et al., 2020; Miyashita et al., 2020). Furthermore, studies from cancer centers and cancer consortiums have evaluated the natural history of COVID19 in larger cohorts of cancer patients and found a mortality rate ranging from 12%-28%(Kuderer et al., 2020; Lee and Naksukpaiboon, 2020; Robilotti et al., 2020). Interestingly, malignancy type did not appear to be a significant predictor of mortality. However, a notable limitation of the existing literature is that many studies utilized small cohorts of cancer patients or did not have a control group of patients without a cancer diagnosis. Furthermore, though our understanding of COVID19 and cancer has continued to evolve, little is known about the impact of cancer history on the risk of developing acute kidney injury (AKI) in COVID19 patients. AKI occurs in an estimated 4.5-8.9% of COVID19 cases(Chen et al., 2020; Yang et al., 2020). AKI increases the risk of developing chronic kidney disease (CKD) (Belayev and Palevsky, 2014), which is concerning for cancer patients as many oncologic therapies may result in renal impairment(Santos et al., 2020). Additionally, in patients with genitourinary malignancies, CKD has been associated with adverse post-operative outcomes(Kumar et al., 2014; Hamano et al., 2017; Ning et al., 2019). 

Our academic medical system includes several hospitals and outpatient clinics throughout the New York City area, which has been one of the world’s most afflicted locations and in turn has treated a large population of COVID19 patients. Accordingly, in the present study, we seek to evaluate COVID19 patients treated within our academic medical system to determine if history of malignancy, both in general and specifically in genitourinary oncology patients, is associated with clinical outcomes, including AKI and mortality. 

## Materials and Methods

*Data Source and Study Design*

Our study received Institutional Review Board Approval (HS#: 20-00875). The study was conducted at a large, New York City based, multi-hospital, academic medical institution. The institution’s Scientific Computing team has been maintaining a regularly updated, deidentified, database of patient-level data from our electronic medical record (EMR) system for encounters related to COVID19. At the time of data extraction for the present study, the database included encounters from March 1st, 2020 through August 10^th^, 2020. 

We included all patients who had a healthcare encounter indicating a positive result for the severe acute respiratory syndrome coronavirus 2 (SARS-CoV2) real-time reverse transcriptase-polymerase-chain-reaction (RT-PCR) assay test during the study period. We identified patient characteristics including demographics, comorbidities (including historical cancer diagnoses), and outcomes from the EMR as well as the location of the encounter. If patients had multiple encounters, an encounter with a positive result was chosen, if applicable. If patients had multiple patient encounters with a positive result, the most severe encounter was chosen. Patients with missing data on any of the variables included in the analysis were excluded. 

*Outcomes*

The primary outcome was all-cause mortality among hospitalized patients (inpatient encounters) with SARS-CoV2 infection. We also evaluated intensive care unit (ICU) admission and AKI among hospitalized patients. Patients with AKI were defined as those with a diagnosis of AKI recorded in their electronic medical record (determined by International Classification of Disease Version 10 codes) during their COVID19 hospitalization encounter. Further, we examined hospitalization among all encounters (inpatient and outpatient). The primary analysis compared outcomes between patients with a history of cancer to those without a history of cancer. Secondary analyses compared outcomes between patients with specific neoplasm locations (kidney, prostate, bladder, lung, hematological, general genitourinary). Oncologic co-morbidities were determined by ICD-10 codes in patients’ problem lists in the EMR. Patients were included in the specific neoplasm location cohorts regardless of whether the ICD-10 codes noted the neoplasm as primary, secondary, or unspecified. Of note, inclusion into the “history of cancer” cohorts was independent of treatment status at the time of hospital admission. That is, the “history of cancer” cohort included all patients with a history of cancer regardless of if they were undergoing active treatment at the time of admission. 

*Potential Confounders*

Because patients with a history of cancer may be different to those without a history of cancer in ways that could be related to the outcomes, the analysis accounted for a comprehensive list of potential confounders. Analyses adjusted for the following variables: age, sex, race, insurance status, smoking status, diabetes, obesity, hypertension, coronary artery disease, chronic kidney disease, atrial fibrillation, asthma, and chronic obstructive pulmonary disorder. Additionally, we performed subgroup analysis on patients with hematologic and lung malignancies given the common pulmonary and hematologic sequalae of COVID19 (Berlin et al., 2020). 

*Statistical Analysis*

We used descriptive statistics and bivariate tests to characterize distributions of covariates across groups for the overall cohort and among those hospitalized. Variables associated with the outcome at a p-value <0.20 in bivariate tests were included in multivariable analyses. We used multilevel logistic regression models for all outcomes to account for clustering of patient outcomes among individual hospitals in the health system. Models estimated odds ratios and 95% confidence intervals (CI).

In sensitivity analyses, we analyzed all-cause mortality and hospitalization using a multilevel Cox proportional hazards model instead. Further, we analyzed AKI and ICU admissions using a multilevel Cox proportional hazards model with mortality as a competing risk. The Cox proportional hazards assumption was verified graphically by generating a plot of the Schoenfeld residuals. In all instances, our primary results were robust and sensitivity analyses yielded similar inferences. Analyses were conducted with R, Version 4.0.0, and used 2-sided statistical tests with a p value < 0.05 as statistically significant in final analyses.

## Results

**Figure 1:**
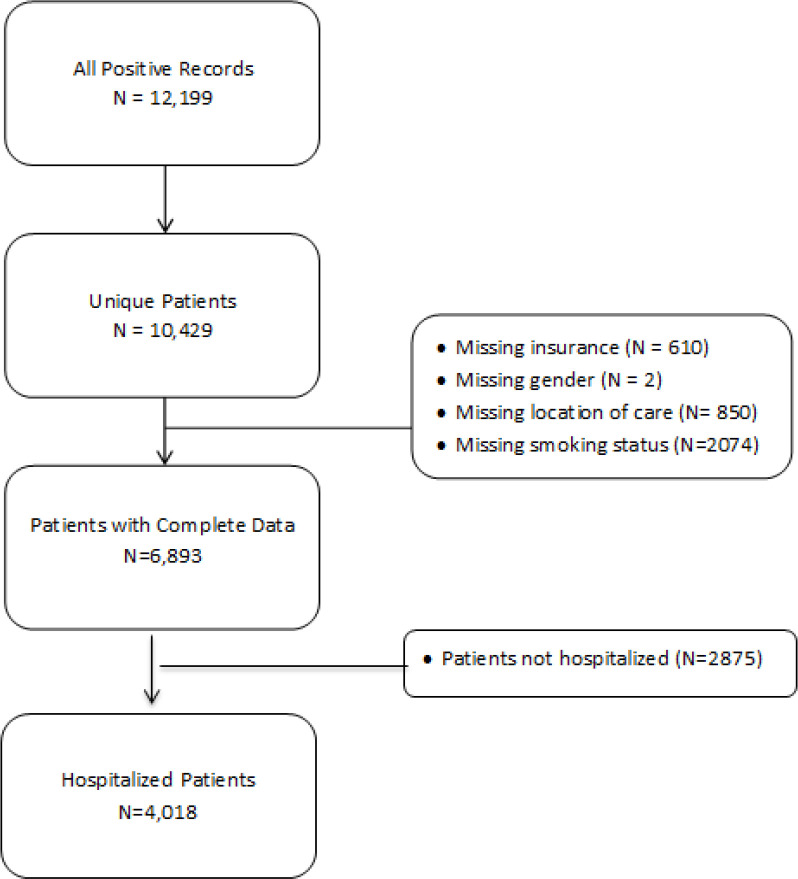
Study Population and Cohort Definition

We identified 10,429 unique patients who met study inclusion criteria. Among these, we excluded 3,596 patients who had missing information leaving us with an overall sample size of 6,893 patients with an inpatient or outpatient encounter (Figure 1). Among these patients, 4,018 were hospitalized, and 720 had a history of cancer. Table 1 reports patient characteristics overall and by mortality status among hospitalized patients (primary analysis). Among the 4,018 hospitalized patients, the average age was 64, 1,844 (46%) were female, 1,060 (26%) were Black, 374 (9%) had a history of cancer, and 1,045 (26%) died. 

Table 2 reports multivariable results for the primary and secondary analyses. For the primary analysis, patients with a history of cancer had 33% increased adjusted odds of mortality compared to patients without a history of cancer (OR = 1.33, 95% CI = 1.05, 1.69). This effect appeared to be largely driven by the increased risk associated specifically with lung cancer. Patients with lung cancer had 144% increased adjusted odds of mortality compared to patients without a history of cancer (OR = 2.44, 95% CI = 1.05, 4.39). Patients with kidney cancer, bladder, cancer, hematological cancer, and all genitourinary cancers did not have an increased mortality risk compared to patients without a history of cancer. Further, patients with prostate cancer had 71% increased adjusted odds of mortality compared to patients with other cancers (OR = 1.71, 95% CI = 1.02, 2.92). Finally, patients with kidney cancer (OR = 0.41, 95% CI = 0.18, 0.95) and those with all genitourinary cancers had lower odds of hospitalization compared to other cancers. We did not observe any other statistically significant effects. 

**Table 1:** Patient Characteristics, Overall and by Mortality among Hospitalized Patients

Characteristics	All (N=4018)	Died (N=1045)	Alive (N=2973)
Any Cancer	374 (9%)	126 (12%)	248 (8%)
Kidney Cancer	15 (0.4%)	4 (0.4%)	11 (0.4%)
Prostate Cancer	76 (2%)	29 (2.8%)	47 (1.6%)
Bladder Cancer	14 (0.3%)	5 (0.5%)	9 (0.3%)
Lung Cancer	14 (0.3%)	6 (0.6%)	8 (0.3%)
Hematological Cancer	54 (1%)	16 (1.5%)	38 (1.2%)
Genitourinary Cancer	106 (2.6%)	39 (3.7%)	67 (2.3%)
Age (mean, SD)	64, 17	73, 12	61, 17
Women	1844 (46%)	444 (42%)	1400 (47%)
Race			
White	1000 (25%)	250 (24%)	750 (25%)
Black	1060 (26%)	293 (28%)	767 (26%)
Other	1958 (49%)	502 (48%)	1456 (49%)
Insurance Status			
Medicare	2105 (52%)	726 (69%)	1379 (46%)
Medicaid	878 (22%)	157 (15%)	721 (24%)
Other	1035 (26%)	162 (16%)	873 (29%)
Smoking Status			
Never	2680 (67%)	647 (62%)	2033 (68%)
Current	1104 (27%)	345 (33%)	759 (26%)
Former	234 (6%)	53 (5%)	181 (6%)
Obesity	410 (10%)	110 (11%)	300 (10%)
Diabetes	1112 (28%)	338 (32%)	774 (26%)
Hypertension	1699 (42%)	523 (50%)	1176 (40%)
Coronary Artery Disease	630 (16%)	222 (21%)	408 (14%)
Atrial Fibrillation	334 (8%)	133 (13%)	201 (7%)
Chronic Kidney Disease	577 (14%)	195 (19%)	382 (13%)
Asthma	257 (6%)	56 (5%)	201 (7%)
Chronic Obstructive Pulmonary Disorder	204 (5%)	74 (7%)	130 (4%)
Acute Kidney Injury	281 (7%)	103 (10%)	178 (6%)

**Table 2:** Odds Ratios and 95% Confidence Intervals from Multilevel Logistic Regression Model

Comparison	Mortality	ICU	AKI	Hospitalization
Cancer vs. No Cancer	1.33 (1.05, 1.69)	1.00 (0.76, 1.30)	0.87 (0.57, 1.34)	0.75 (0.55, 1.06)
Kidney Cancer vs. No Cancer	0.65 (0.21, 2.08)	0.61 (0.13, 2.73)	0.54 (0.07, 4.01)	0.41 (0.18, 0.95)
Kidney Cancer vs. Other Cancer	0.55 (0.16, 1.95)	0.58 (0.12, 2.85)	0.79 (0.10, 5.98)	0.83 (0.35, 1.97)
Prostate Cancer vs. No Cancer	1.15 (0.71, 1.86)	0.99 (0.57, 1.71)	0.88 (0.38, 2.07)	0.74 (0.47, 1.20)
Prostate Cancer vs. Other Cancer	1.11 (0.90, 1.38)	1.26 (0.63, 2.51)	0.82 (0.31, 2.18)	1.71 (1.02, 2.92)
Bladder Cancer vs. No Cancer	0.87 (0.29, 2.65)	0.66 (0.14, 2.99)	**	0.50 (0.19, 1.32)
Bladder Cancer vs. Other Cancer	0.88 (0.27, 2.92)	0.75 (0.16, 3.55)	**	1.20 (0.45, 3.24)
Lung Cancer vs. No Cancer	2.44 (1.05, 4.39)	0.99 (0.27, 3.60)	1.16 (0.15, 8.69)	0.85 (0.54, 1.28)
Lung Cancer vs. Other Cancer	1.53 (0.51, 4.64)	0.79 (0.20, 3.09)	0.62 (0.08, 4.90)	0.98 (0.72, 1.41)
Hematological Cancer vs. No Cancer	1.32 (0.72, 2.43)	1.18 (0.62, 2.24)	0.98 (0.34, 2.78)	1.01 (0.65, 1.78)
Hematological Cancer vs. Other Cancer	1.14 (0.82, 1.57)	1.03 (0.77, 1.50)	0.92 (0.41, 1.83)	1.10 (0.68, 1.96)
Genitourinary Cancer vs. No Cancer	0.99 (0.61, 1.62)	1.14 (0.67, 1.94)	0.52 (0.19, 1.43)	0.62 (0.39, 0.97)
Genitourinary Cancer vs. Other Cancer	0.91 (0.51, 1.63)	1.58 (0.82, 3.05)	0.44 (0.15, 1.31)	1.14 (0.77, 1.71)

**, Not enough events to analyze; AKI, Acute Kidney Injury; ICU, intensive care unit admission

## Discussion

For cancer patients, navigating the COVID19 pandemic has brought unique challenges. Balancing the risk of contracting COVID19 against the risk of delaying oncologic care has thus far been informed by the natural history of malignancies, while the natural history of COVID19 in cancer patients remains an area of active research. Prior studies have attempted to define clinical outcomes in cancer patients with COVID19 (Berenguer et al., 2020; Giannakoulis et al., 2020; Kuderer et al., 2020; Lee and Naksukpaiboon, 2020; Liang et al., 2020; Robilotti et al., 2020). One of the first such studies evaluated 1590 patients from China with COVID19, of which 18 patients had a history of malignancy, and found that a history of malignancy was associated with poor clinical outcomes(Liang et al., 2020). Another population based study from Spain evaluated 4,035 consecutively hospitalized patients with COVID19 and found that presence of an active malignancy (359 patients) was an independent predictor of mortality (Berenguer et al., 2020). A study of 5,366 COVID19 patients in New York City early in the pandemic found that a history of cancer (334 patients) conferred a greater risk of intubation, though not a greater risk of death (Miyashita et al., 2020). 

Studies from cancer centers have evaluated the natural history of COVID19 in larger cohorts of cancer patients. A prospective observational study of 800 cancer patients in the United Kingdom with COVID19 reported a 28% mortality rate. Interestingly, the risk of mortality was independent of cancer type (Lee and Naksukpaiboon, 2020). However, in a cohort of 423 patients treated at a cancer center New York City, the mortality rate was notably lower at only 12% (Robilotti et al., 2020). Indeed, a multi-institutional study of 928 patients with COVID19 and cancer throughout the US and Canada found a mortality rate of 13% (Kuderer et al., 2020). Malignancy type was not a significant predictor of mortality. Notably, none of these studies compared outcomes to those of patients without cancer. One meta-analysis which included 46,499 COVID19 patients, including 1,776 of whom had cancer, found that malignancy is associated with an increased risk of death and intubation (Giannakoulis et al., 2020). However, in a subgroup analysis of patients > 65, there was no increased risk of mortality from COVID19 amongst cancer patients. 

Though our understanding of COVID19 and cancer has continued to evolve, little is known about the impact of a history of cancer on the risk of developing AKI in COVID19 patients. Indeed, to our knowledge, no such studies have evaluated AKI as an outcome. Furthermore, many of the existing studies had relatively small samples of cancer patients or did not have a non-cancer patient control. To this end, in the present study, we have a conducted a retrospective analysis of a large cohort of patients hospitalized with COVID19 in the New York City area to evaluate the impact of malignancy on patient outcomes, including AKI, with a focus on patients with genitourinary malignancies. 

Notably, our cohort represents approximately 4% of positive cases in New York City and 7% of deaths in New York City as of August 10^th^, 2020. Approximately 7% of all hospitalized COVID19 patients in our cohort had a diagnosis AKI, an incidence concordant with the 4.5%-8.9% range reported in the literature(Chen et al., 2020; Yang et al., 2020). Notably, there was no increased risk of AKI amongst patients with a general history of malignancy, or amongst patients with a history of genitourinary malignancy. This key finding informs us that patients with a history of cancer are not more likely to develop AKI when hospitalized with COVID19. AKI is of particular concern for cancer patients as AKI is associated with the subsequent development of CKD, and CKD is associated with adverse post-operative outcomes in patients with prostate, kidney, and bladder cancer(Kumar et al., 2014; Hamano et al., 2017; Ning et al., 2019). Furthermore, CKD may reduce the oncologist’s clinical armamentarium as several chemotherapeutic agents are nephrotoxic and optimal surveillance imaging often requires iodinated contrast enhancement(Santos et al., 2020). 

Regarding mortality, overall, approximately 26% of patients who were hospitalized died, and amongst hospitalized cancer patients, approximately 34% of patients died. History of malignancy conferred a statistically significant 33% increased relative risk of mortality among hospitalized patients. However, upon analysis of malignancy sub-types, we found that this increased mortality risk appears to be driven by patients with pulmonary neoplasms. Indeed, patients with lung tumors had a greater than two-fold risk of death. On the contrary, patients with prostate, bladder, kidney, or general genitourinary malignancies were not at greater risk of mortality compared to the patients without cancer. Thus, though patients with malignancy may be at higher risk for mortality, this heightened risk does not appear to apply to patients with genitourinary malignancies. Interestingly, though patients with lung cancer and cancer in general had higher mortality rates, they did not have higher rates of ICU admission. This may be related to the shortage of ICU beds during the local peaks of the pandemic (Sprung et al., 2020). 

In addition to evaluating outcomes amongst hospitalized patients, we evaluated risk of hospitalization amongst all SARS-CoV-2 positive patients. Overall, we found no increased risk of hospitalization amongst patients with a history of cancer compared to those without a cancer diagnosis. Interestingly, we found an overall lower risk of hospitalization amongst patients with a history of genitourinary malignancy compared to patients without cancers. Indeed, on subgroup analysis this lower risk of hospitalization appears to be driven by patients with a history of kidney cancer. The explanation for this trend is unclear. However, one possibility is that the renin-angiotensin-system (RAS) is altered in patients with renal cancer. Indeed, SARS-CoV-2 binds via the angiotensin-converting enzyme 2 receptor (Zhang et al., 2020) and previous research has shown that patients with renal cancer have differential expressions of RAS enzymes (Larrinaga et al., 2010). Ultimately, further research is required to understand this pattern. 

Our study has several notable limitations. We conducted a retrospective analysis of an existing database rather than a prospective study. Furthermore, given the nature of the database and present study, we did not have data regarding tumor stage and grade and accordingly could not control for these variables. For example, in defining a patient as having a history of “lung cancer,” we included all patients with a diagnosis code indicative of a lung neoplasm, including secondary and unspecified lung neoplasms. Given that the lung is a common site of metastatic spread, our lung cancer cohort may have included patients with more advanced malignancies which may in turn be contributing to the higher mortality rate observed. Patients also were not separated by those with active malignancy and those on surveillance. Indeed, we did not adjust for stage or any other cancer-specific severity variable as this because it would not be appropriate or feasible to include stage in the multivariable model for the primary analyses (cancer vs no cancer) because stage of cancer is a variable that only applies to those with cancer and not to those without cancer. Though we considered adjusting for cancer stage in our analyses comparing individual cancers, the heterogeneity of staging between different cancer types may have introduced measurement error and associated miscalculation. A further limitation was that amongst the individual genitourinary malignancies analyzed (prostate, bladder, kidney), our sample sizes were small. Additionally, regarding mortality, our outcome was all-cause mortality, and thus it is unclear if the differences in mortality are being driven by COVID19 or other factors. While COVID19 specific mortality may have allowed for greater insight, our rapidly evolving understanding of the natural history of COVID19 would make such mortality determinations largely subjective. Despite these limitations, we believe our study provides an important adjunct to the existing literature.

In conclusion, overall, our study findings suggest that patients with a history of cancer who are hospitalized with SARS-CoV-2 are not at greater risk for AKI, though they are at higher risk for mortality as compared to hospitalized patients without a cancer diagnosis. The increased risk in mortality appears driven by patients with pulmonary neoplasms. Patients with genitourinary malignancies do not appear to be at higher risk for mortality or for AKI compared to those without malignancy. 

This finding is of great clinical importance as we navigate the “new-normal” phase of the COVID19 pandemic. Healthcare providers must guide patients through weighing the risks of delaying therapy against the risks of contracting COVID19 in the nosocomial setting. Understanding that the risk of adverse outcomes in patients with histories of genitourinary malignancy is similar to that of the general public may help guide patients through this challenging and anxiety provoking risk-benefit analysis. Further study with a larger cohort of genitourinary malignancy patients in a prospective manner is warranted. 
